# Children’s mental time travel into the future: a functional perspective

**DOI:** 10.1098/rstb.2023.0399

**Published:** 2024-09-16

**Authors:** Gladys Ayson, Cristina M. Atance

**Affiliations:** ^1^ School of Psychology, University of Ottawa, Ottawa K1N 6N5, Canada

**Keywords:** mental time travel, future-thinking, cognitive development, naturalistic approach

## Abstract

Children’s episodic future-thinking is typically assessed using experimental tasks that measure whether children select an item with future utility. Although these tasks—inspired by Tulving’s seminal ‘spoon test’ (Tulving E. 2005 Episodic memory and autonoesis: uniquely human? In *The missing link in cognition: origins of self-reflective consciousness* [eds HS Terrace, J Metcalfe], pp. 3–56. Oxford, UK: Oxford University Press. [doi:10.1093/acprof:oso/9780195161564.001.0001])—are passed around age 4, they tell us little about the functional significance of children’s episodic future-thinking in their day-to-day lives. We highlight how a naturalistic approach can shed light on this issue, and present a small study where we recruited mothers to report on their children’s (*N* = 12, 3- and 4-year-olds and 6- and 7-year-olds) future-thinking over a 7-day period. We used a thematic analysis to understand why children express future thoughts and derived the following themes: (1) expressing future desires and/or intentions, (2) future-oriented information-seeking, (3) connecting present actions with future outcomes, and (4) predicting future mental/physiological states. We compare these themes with recent accounts of the functional significance of future-thinking in adults and conclude that children largely express their future-thinking verbally to request information or support from their parent—likely because they do not yet possess enough control/autonomy to independently act for their own future. Our findings both complement and extend an experimental approach and further elucidate the functional significance of mental time travel in children.

This article is part of the theme issue ‘Elements of episodic memory: lessons from 40 years of research’.

## 1. Introduction

People think about the future daily, and these thoughts affect how they feel and behave in the present. Episodic future-thinking, in particular, involves mentally projecting the self forward in time to pre-experience personal events [1]. A proposed taxonomy of episodic future-thinking suggests four modes: simulation, prediction, intention and planning [2]. Moving through these modes may bring an individual closer to engaging in behaviour with the future in mind. For example, I can simulate spending time outdoors on my vacation in Portugal, which leads me to predict being in rainy weather based on the time of year. In response to this prediction, I intend to pack appropriate clothing for my trip, and, when packing, I plan for the rainy weather by packing my raincoat and rainboots in my suitcase. Planning is often necessary when carrying out intended behaviours because it involves converting a goal into ‘actionable’ steps [2]. Indeed, contemplating future events is argued to be adaptive precisely because it allows us to take action now to address possible futures [3–6]. My thought about my upcoming vacation in Portugal is expressed through a concrete action that helps prepare me for what lies ahead (i.e. packing raingear in my suitcase). Of course, adults also ‘express’ their future thoughts through language and conversation. For example, I may tell my friend about my upcoming vacation and she, in turn, give me tips on travelling efficiently.

Our thoughts about the future, including those reflected in our actions and talk, serve numerous functions in our daily lives. For example, packing my suitcase for Portugal highlights what has been termed a ‘directive’ function of future-thinking that supports goal pursuit, problem-solving, planning and decision making [4,5,7,8]. Thinking about the future can also serve an ‘emotion regulation’ function; specifically, thinking about positive future events (e.g. my upcoming vacation) can upregulate positive emotion in the present [5], and thinking about negative future events (e.g. getting through airport security) can prepare me for potential adverse feelings [4]. Finally, thinking about the future contributes to forming and exploring one’s identity, and sharing future thoughts with others can strengthen social bonds [5,8]. For example, discussing my trip itinerary with my friend increases our feelings of closeness. In sum, adults express their future thoughts in a variety of ways (i.e. language and action), and these expressions serve numerous functions in their daily lives. Despite the prevalence of future-thinking in adults, along with its numerous pragmatic benefits [9], we know little about the functions of future-thinking in children’s daily lives. The main reason for this gap in our knowledge is that research about children’s future-thinking has primarily been conducted using single tasks that often direct the child to consider the future, rather than children, themselves, functionally engaging their future thought.

Our over-arching goal in the current article is thus to explore both how children express future thoughts in their daily lives and the functional role these thoughts may serve. We begin by briefly describing how children’s episodic future-thinking has traditionally been measured by focusing on what have come to be known as ‘spoon’ tasks. A key argument we make is that because these tasks have exclusively been implemented in the laboratory, they cannot speak to the diverse ways and reasons children express their future-thinking. To address these issues, a naturalistic approach is needed, and we subsequently outline a new methodology that recruits parents to report on their children’s expressions of future-thinking.

### (a) Measuring children’s episodic future-thinking: spoon tasks

One of the most popular means of measuring children’s episodic future-thinking has been heavily inspired by Tulving’s ‘spoon test’ [10, p. 44]:

a young girl dreams about going to a friend’s birthday party where the guests are served delicious chocolate pudding, her favorite. Alas, all she can do is to watch other children eat it, because everybody has to have her own spoon, and she did not bring one. So the next evening, determined not to have the same disappointing experience again, she goes to bed clutching a spoon in her hand.

Because it is the girl’s action of retrieving a spoon that reflects her thought about the future, Tulving argued that the ‘test’ is particularly well suited to assess whether non-human animals and young children can think episodically about the future. Tulving’s test (see also earlier proposals in [11,12]) has been translated into experimental ‘spoon’, ‘two-room’ or ‘item-choice’ tasks that typically entail children encountering a ‘problem’ in which they lack a specific item (e.g. a key to unlock a box with stickers inside). Later, in a different location, children are presented with a set of items (including a key) and are asked to select one to bring with them to the original setting. Selecting the appropriate item is taken as evidence of children’s capacity to anticipate and prepare for the future event. Numerous studies have shown that, around age 4, children pass these tasks [13–15].

However, as we have recently argued [3], these tasks do not require children to *independently* act with the future in mind because children are prompted—in the form of an explicit test question—to perform a future-oriented action. In this sense, such tasks deviate from Tulving’s proposed test in which the girl acts of her own volition, or ‘spontaneously,’ to obtain a spoon. A second, more fundamental issue that we tackle in the current article is that such tasks do not capture the breadth of functions future-thinking may serve in young children’s everyday lives. Indeed, spoon tasks are best suited to measuring the ‘directive’ function of future-thinking, leaving other functions unassessed.

### (b) The need for a naturalistic complement to experimental tasks

There has been a recent push for more naturalistic research in developmental psychology [16,17]. For example, Dahl [16] points out that the term ‘naturalistic’ was used in fewer than 5% of studies published in seven developmental journals between 1967 and 2017. This is non-trivial given that Dahl (and other) authors argue that, without such work, it is impossible to capture children’s ‘lived experience’—specifically, the everyday scenarios through which they develop their cognition [17]. We echo these sentiments and argue that an experimental approach alone will not enable us to understand the diverse ways in which children express their future-thinking and the various uses to which they apply this form of thought.

For example, consider whether a 3-year-old (who may fail the spoon task) typically uses their future thoughts in a directive manner (as do adults). It is possible that they do; for instance, this 3-year-old may be capable of episodically projecting into the future to think about an upcoming playdate with their friend and this thought might motivate them to pack a toy to bring along (i.e. directive function). Alternatively, this 3-year-old may use their future thoughts quite differently from the way adults do. For example, thoughts about playing with their friend could lead them to seek assistance (e.g. ‘Can you help me find a toy?’) or information (‘Is it going to rain tomorrow?’) from an adult. Our goal is not to determine whether spoon tasks assess children’s capacity to think about the future ([3], for an in-depth critique); rather, our main point is that they, and other experimental measures, cannot capture how children express their future thoughts in daily life and, hence, the possible functions they serve.

Addressing these issues requires adopting a naturalistic approach in which children are observed outside the laboratory. The advantages of such an approach are twofold. First, it can shed light on the functional significance of mental time travel from its earliest emergence (thus complementing what we know from experimental tasks). Second, it can lead to the creation of experimental tasks that better reflect the kinds of situations, motivations and challenges children experience in their daily lives (an issue to which we return in §5). Our aim is to use this approach to provide a preliminary account of how children express their future thoughts and the functions these serve.

### (c) A naturalistic investigation of children’s future-thinking

The various functions of future-thinking in adults have been identified using experience sampling and self-report studies in which participants are asked to monitor and report their own naturally occurring future thoughts and perceived functions [4,5,7–9]. While young children have been shown to ‘report’ on their spontaneous memories in a laboratory setting (e.g. [18–20]), they may find it difficult to monitor (and report on) their thoughts during their highly stimulating daily lives. Accordingly, we recruited parents of 3- and 4-year-olds and 6- and 7-year-olds to identify and report ‘instances’ of their children’s future-thinking over the course of one week. We use the term ‘instances’ to reflect both children’s verbal statements about, and actions for, the future. Our methodological approach was inspired by previous naturalistic research on children’s memory [21] and adults’ spontaneous future-thinking [22]. To determine the feasibility of our novel methodology, we ran a small proof-of-concept study with a sample of *N* = 12 children.

We hypothesized that parents would report more instances of future-thinking in older (ages 6 and 7), as compared with younger (ages 3 and 4), children, given the well documented improvements in this form of thought over early-to-middle childhood (see [23] for a review). Moreover, we predicted that the parents of older, as compared with younger, children would report more future-thinking actions, reflecting children’s ability to proactively use their future-oriented thoughts to inform and direct their behaviour [3]. Although we made no specific hypotheses about the potential functions of children’s future-thinking, we nonetheless expected that, as with adults, at least some of the instances reported by parents would reflect a ‘directive’ function.

## 2. Methods

### (a) Participants

We recruited 11 English-speaking families from the University of Ottawa developmental laboratories’ database. All consenting parents were mothers. Mothers with more than one eligible child were given the option of tracking them, and three agreed to track two of their children simultaneously. One mother was subsequently removed from the analysis for being unable to maintain tracking. This mother was tracking a 6-year-old and a 7-year-old. Another mother stopped tracking her 7-year-old owing to an overwhelming number of instances. This resulted in a final sample of 12 children (three 3-year-olds, three 4-year-olds, four 6-year-olds and two 7-year-olds) and 10 mothers. To examine potential age-related differences, we compared the data of our younger (3- and 4-year-olds; *n* = 6) and older (6- and 7-year-olds; *n* = 6) age groups.

### (b) Procedure

#### (i) Introductory meeting

Mothers met with the first author on Zoom to learn more about the study and its requirements. They were told that they needed to identify and report their child’s (or children’s) future-thinking statements and future-thinking actions. Future-thinking statements were defined as verbal utterances about the future (e.g. asking the parent to pack a snack for an outing tomorrow) and need not include future-oriented temporal terms (e.g. tomorrow, later). Future-thinking actions were defined as any action performed by the child that the parent believed was executed with the future in mind (e.g. setting aside a drawing to finish later). Mothers were encouraged to include all instances (even those they were unsure of) to capture a wide breadth of instances (which would later be examined by the researchers). They were instructed to only report ‘spontaneous’ instances of future-thinking; that is, instances in which their child either did or said something ‘on their own’, rather than being told/asked to do so by someone else. At the end of the meeting, mothers decided whether they were interested in participating, and all agreed. Verbal consent was then obtained and video-recorded on Zoom, and mothers chose the dates for their tracking period.

#### (ii) Tracking period

Tracking dates were scheduled between July and August 2021 and lasted one week (7 days). Our study was conducted during a time when individuals were facing institutional closures and societal changes resulting from the COVID-19 pandemic. While outdoor facility restrictions had mostly been lifted in Canada at this time, we intentionally scheduled tracking dates to be during the summer when children were not in school. During the informational meeting, we asked parents whether their children were engaged in any online and/or in-person activities during their time of tracking. Only two children were engaged in virtual activities (Spanish class and taekwondo). Three children attended in-person daycare or nursery school, and five children attended in-person sports or music lessons.

When mothers observed an instance of their child’s future-thinking (i.e. future-thinking action or statement), they were instructed to write down keywords to help them remember it and where/when it occurred. Mothers decided where and how to document their keywords, with most doing so on their phones or in a notebook. These keywords were not analysed.

Mothers were instructed to complete a questionnaire for each future-thinking action or statement that they witnessed. The questionnaire asked mothers to describe the instance and what was happening at the time (i.e. surrounding context) in as much detail as possible. Mothers were asked to report where (e.g. inside their home) and what time of day (i.e. morning, afternoon or evening/night) the instance occurred. Mothers were encouraged to complete the questionnaires at the end of each tracking day but could ultimately do so at their convenience. However, mothers could only schedule their wrap-up meeting once they had sent their completed questionnaires to the research team.

#### (iii) Wrap-up meeting

Following their tracking period, mothers scheduled a wrap-up meeting with the first author to discuss their experience. They were asked whether their tracking period was a ‘typical’ or ‘atypical’ week for the family. Seven out of 10 mothers reported their tracking days to be ‘typical’, and the remaining three reported atypical tracking days because of a family member being sick (*n* = 2) or a family trip occurring while tracking (*n* = 1). Mothers also reported what percentage of their child’s future-thinking they felt they ‘missed’ while tracking. The average percentage reported across parents was 33.5% (range 5–65%).

## 3. Analysis

### (a) Reflexive thematic analysis

A reflexive thematic analysis (TA) was conducted by the first author to identify, analyse and interpret patterns across reported instances. The guiding research question was ‘Why do children express their future-thinking in their daily lives?’ We chose reflexive TA because it is a data-driven approach that utilizes the researcher’s field knowledge to flexibly derive meaning from the data. Our methodology was guided by Braun & Clarke’s [24,25] six phases, described below.

#### (i) Phase 1: data familiarization

The researcher first entered all instances into an Excel file, and read, and re-read each data point (i.e. instance) to gain familiarity with the data. General notes were also made regarding initial, surface-level patterns.

#### (ii) Phase 2: data coding

The researcher began coding by assigning descriptive or semantic codes to each data point (e.g. child asking ‘“Can I …?” questions’, child making ‘“I will …” statements’). In subsequent pass-throughs, the data was re-coded incorporating a deductive or ‘researcher-driven’ viewpoint by grouping together codes with similar latent meaning (e.g. ‘stating what will happen in the future’, ‘establishing a timeline/sequence of events’). On the fourth cycle of coding, the researcher printed out the data points (and their current codes) and visually sorted them.

#### (iii) Phase 3: initial theme generation

The researcher further grouped together codes that shared a patterned ‘meaning’ (e.g. expressing a want or desire) related to the guiding research question. Groups were re-sorted when data points or codes could be assigned to more than one theme.

#### (iv) Phase 4: theme development and review

Themes from phase 3 were then reviewed by reading each instance within each theme. The researcher used guiding questions such as ‘Can I identify the boundaries of this theme?’, ‘Is the data within this theme too diverse or wide-ranging?’ and ‘Is there enough data or evidence to support this theme?’ to support the theme structure. The researcher navigated between this phase and phase 3 until all instances supported their assigned theme and not any other.

#### (v) Phases 5 and 6: theme refining, defining, naming and write-up

The researcher wrote a brief definition of the finalized themes to highlight the central organizing concept, the theme’s boundaries and specificities and what the theme contributes to the overall research question. These definitions are summarized in table 1.

**Table 1.** Summary of themes.

theme	definition	examples from our data
theme 1: expressing future desires and/or intentions	expressions of intention or ‘desire’ for something to happen in the future	child asked to call grandma after breakfast ‘Next time we see a cat that doesn’t have a home, can we take him?’
theme 2: future-oriented information-seeking	questions about what will happen in the future or when a future event will occur	‘Will I need braces when I’m older?’ ‘When can we go to daycare?’
theme 3: connecting present actions to future outcomes	expressing (through statements or actions) knowledge that something done now will impact the future	child grabbing toys for him and sister to play with in the car ‘I don’t want to go swimming today because the sun is not out and it will be too cold’
theme 4: predicting future mental and physiological states	statements or questions about what the child (or somebody else) will feel like (or do) in the future	a child says that on her birthday she will be too excited to sleep in and will probably wake up at 6.00 ‘Won’t everyone be surprised that they have to start with black when playing Go?’

## 4. Results

### (a) Total instances

The 10 mothers (*N* = 12 children) reported a total of 244 instances. After extensive discussion, the authors determined that 24 of mothers’ reported instances were not future-oriented and were thus excluded from analyses (e.g. a child saying ‘Pretend I’m 16 years old’ while playing a game). An additional five instances were excluded for having been prompted by an ongoing conversation, leaving a total of 215 analysable instances.

Mothers reported an average of 17.9 future-thinking instances over the one-week tracking period (range = 4–70). One mother reported 70 valid instances, which was over three times the number reported by the other mothers. However, because this was a new methodological approach with a small sample, we opted to keep this participant in our analyses. Table 2 summarizes the distribution of valid instances across participants. The instances occurred in the home (75.4%), car (14.9%), at a public establishment (1.9%), at a grandparent’s house (1.4%) or at an outdoor location outside the home (6.5%). The total number of reported instances did not differ significantly between younger (median = 12) and older (median = 18.5) children, *U* = 27*, z =* 1.451*, p* = 0.180.

**Table 2.** Number of reported instances. Individuals marked with * are siblings; individuals marked with † are siblings.

age group	age	reported Instances	statements	actions
younger	3^*^	12	12	0
younger	3^*^	13	12	1
younger	3^†^	12	12	0
younger	4	17	16	1
younger	4	8	7	1
younger	4	4	4	0
older	6	21	21	0
older	6^†^	16	14	2
older	6	70	69	1
older	6	6	5	1
older	7	24	21	3
older	7	12	11	1
**total**		**215**	**204**	**11**

A Friedman test showed that the proportion of reported instances did not significantly differ by tracking day, *χ* ^2^(6) = 9.455, *p* = 0.150, but did significantly differ by time of day (morning, afternoon and evening/night; note that six instances did not have a reported time of day), *χ* ^2^(6) = 10.978, *p* = 0.004. A *post hoc* analysis with a Bonferroni correction (*p* = 0.017) revealed a higher proportion of instances in the morning (*M* = 0.43) compared with the evening (*M* = 0.19), *p* = 0.005.

**Figure 1.**
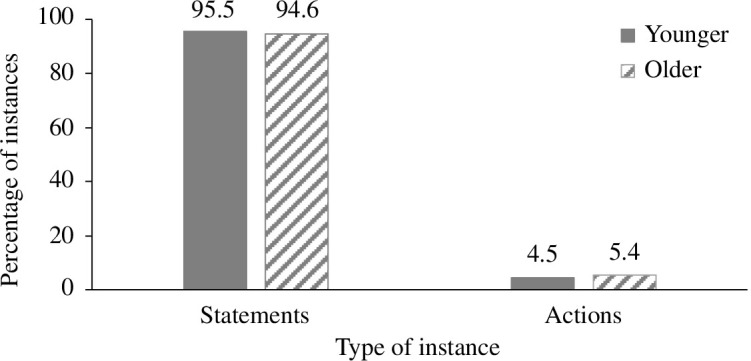
Percentage of statements and actions by age group.

Whereas 94.9% of children’s reported instances were statements (range = 83–100%), only 5.1% were actions (range = 0–17%). Percentage of actions and statements per age group are shown in figure 1. Parents reported a significantly higher proportion of statements than actions, *t*(11) = 24.669, *p* < 0.001, and this proportion did not differ as a function of age, *t*(10) = 1.224, *p* = 0.249.

### (b) Thematic analysis

We derived four themes which we describe below and summarize in table 1. Six instances (five statements and one action) were unclassified because they did not fit any theme.

#### (i) Theme 1: expressing future desires and/or intentions

This theme included expressions of ‘want’ or ‘desire’ for something to happen in the future. This was the most prevalent theme in our data, comprising 98 instances (45.6%), all of which were statements. Specifically, this included children requesting permission to have or do something (e.g. ‘Next time can we make a fort?’, ‘Can we go there when the virus is over?’) and expressions of intentions (e.g. ‘When I’m a daddy I’m going to make s’mores the first day’, ‘Tomorrow I will share it with you’). Instances in this theme resemble the ‘intention’ mode of thinking in adults [2], in which future goals or wants are expressed. Interestingly, 52 of the 98 instances (53%) were requests for the parent to give or do something, which may reflect children’s reliance on parental permission or support to fulfil their intentions.

#### (ii) Theme 2: future-oriented information-seeking

This theme pertained to children seeking information about what, whether or when a future event would occur. This was the second most prevalent theme, with a total of 60 instances (27.9%). All were statements, specifically questions, which were sorted into this theme on a largely semantic basis, in that they were questions about ‘what’, ‘when’ or ‘if’ something would happen.

#### (iii) Theme 3: connecting present actions to future outcomes

Instances in this theme demonstrated knowledge that something done now would impact the future. There were 43 instances in this theme (20%): 33 statements and 10 actions. To qualify under this theme, children’s statements/actions needed to reflect a causal connection between the future and their current statement/action. Statements may also contain an intention or prediction; however, to be categorized into theme 3, and not theme 1 or 4, the child needed to explicitly identify a future consequence or outcome. Instances in this theme reflect the ‘planning’ mode of future-thinking [2], in which actionable steps in the present are identified towards a future outcome or goal. Interestingly, similar to theme 2, a large portion of the instances in this theme (17 instances, 39.5%) were questions, requests or reminders for the parent to act (e.g. a child asking their mother to call their friend’s parent to arrange a playdate). Again, this signals children’s dependence on parents to bring about a desired outcome.

#### (iv) Theme 4: predicting future mental and physiological states

This theme represented predictions about what the child (or somebody else) would feel like (or do) in the future. Although only eight instances (3.7%) fell into this theme, and all were statements, it was retained because of the previous developmental literature on predicting future physiological and mental states (e.g. [26,27,]) and literature on the functionality of prediction in adults (i.e. ‘affective forecasting’ [6,28,29]; and ‘prediction’ as a key mode of prospection [2]). Thus, it is possible that, with a larger sample, this theme would be more prevalent.

**Figure 2.**
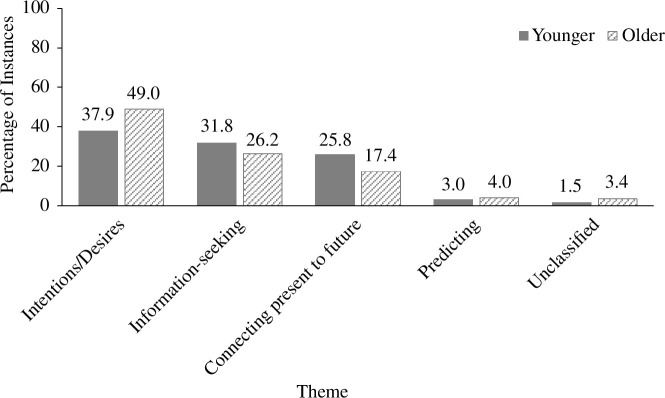
Percentage of instances across themes for each age group.

A series of Mann–Whitney *U*-tests revealed that the proportions of instances categorized into theme 1 (*U* = 19.5, *z* = 0.245, *p* = 0.818), theme 2 (*U* = 15.5, *z* = −0.402, *p* = 0.699), theme 3 (*U* = 16.5, *z* = −0.243, *p* = 0.818) and theme 4 (*U* = 22, *z* = 0.714, *p* = 0.589) did not significantly differ as a function of age group. The percentages of instances that were categorized into each theme across age groups are shown in figure 2.^1^ Although reliability coding is not recommended in a reflexive TA—because themes are developed and not ‘found’ in the data [25]—we provided a second coder with our theme descriptions and 33% of our instances to determine whether they would classify these instances similarly into our corresponding themes. This second coder had 77% agreement with our original theme sorting, reflecting a substantial level of agreement, *κ* = 0.662, *p* < 0.001.

## 5. Discussion

Children’s daily experiences have received little attention in studies about their cognitive development [17]. Research on children’s future-thinking is no exception as it has predominantly focused on developing structured, experimental tasks which have additional cognitive demands (e.g. language, memory and unfamiliarity) that may partly mask children’s future-thinking abilities. To our knowledge, this study is the first to investigate the various functions of children’s future thoughts in their everyday lives. Our findings contrast with evidence from experimental tasks by showing that even 3-year-olds demonstrate sophisticated forms of future-thinking in their own homes. It may be that experimental tasks underestimate children’s future-thinking abilities or that, in a naturalistic context, individual differences are more pronounced than age-related ones.^2^

### (a) Reported instances

On average, parents reported between two and three future-thinking expressions a day. This amount is comparatively much smaller than in adults, who report an average of 59 future thoughts a day [7]. This difference may be both developmental and methodological. With respect to the latter, adults self-report on their own cognition, whereas our study uses a third-party observer (i.e. child’s parent). Therefore, we are only obtaining instances of future thoughts that were expressed through the child’s statements or behaviours, and thus we cannot be certain that the parent caught every future-thinking expression their child made during the tracking period. In fact, on average, parents suspected they missed about one-third of their child’s future-thinking while tracking. Owing to these differences in methodology, comparisons between our findings and adult self-report findings should be made with caution.

We found no age differences in the proportion of statements and actions that mothers reported nor in their categorization by theme. However, these null findings should be interpreted cautiously as they could be due to our small sample size. That said, our lack of age differences is consistent with a recent diary study [30] using a larger sample that reported no significant age differences in 2- to 6-year-olds' everyday prospective memory successes and failures. Although additional work is needed, age differences in future-thinking using experimental tasks do not seem to be reflected in the naturalistic data. Alternatively, at least in our study, it is possible that mothers of younger children spent more time with them than mothers of older children, which increased the number of reported instances. This could be mitigated by controlling for the number of hours the tracker spent with their child in subsequent analyses. While we asked mothers to report the number of hours they spent with their child each tracking day, we could not use this value in our analysis because some mothers wrote ‘all day’ rather than providing a specific number.

We found that mothers reported fewer future-thinking instances in the evening compared with the morning, which is consistent with findings in adults, where thoughts about the future (and the past) decrease throughout the day, while thoughts of the present increase [9]. It may be that future-thinking is optimized in the morning; perhaps getting ready for the day (e.g. picking out an outfit) primes children to think about upcoming events, whereas in the evening, families may be discussing and reminiscing on the day’s events. It is also possible that the decrease in reported instances throughout the day is due to reporter fatigue. Mothers may tire by day’s end, which impacts their vigilance and, in turn, the number of instances they observe. In contrast, they may feel more energized in the morning, which enables them to identify more instances. However, mothers’ reported number of instances did not significantly differ across tracking day, suggesting that our methodology was feasible for them over the 7-day period.

### (b) Actions versus statements

Of the 244 instances analysed, only 11 were actions. Given the small number of reported actions overall (5.1%), behavioural expressions of future thoughts may not be typical for younger nor older children. Instead, children may be more accustomed to expressing their future-thinking in ‘words’, not ‘action’. If so, this suggests that, despite behavioural measures being the favoured option in developmental research (e.g. because they do not rely on linguistic abilities [6,12]), children themselves may mostly express their thoughts about the future verbally.

Notably, a prevailing way in which children expressed their future thoughts was in the form of questions and requests to their parents (135 instances; 66% of statements). This suggests that children seek their parents to validate their future predictions and carry out their intentions. Consistent with this claim, the high prevalence of theme 1 instances (‘future-oriented information-seeking’; 27.9%) coupled with the low prevalence of theme 4 instances (‘predicting future mental and physiological states’; 3.7%) suggests that children require information about the future before they can make predictions about it. Reliance on parental intervention may in fact ‘block’ children from progressing their future-oriented thoughts into actionable ‘plans’ because they are not in control, capable and/or aware of the necessary steps required to do so [31]. Indeed, 53% of theme 1 instances (‘expressing future desires or intentions’) were requests to the parent (e.g. ‘Can we go there when the virus is over?’). Furthermore, even though theme 3 (‘connecting present actions to future outcomes’) reflects children’s actionable planning, nearly half of those cases (39.5%) also required parental assistance (e.g. a child asking their parent to call a friend’s mother to set up a playdate).

It is important to recognize, however, that parents may find it easier to identify (and report) statements made directly to them. Accordingly, our methodology may inflate the number of requests, and overall statements, reported. It might also be challenging for parents to detect future-oriented behaviours and statements a child makes to others because these could be done covertly and/or when their parent is not actively engaged with them. This may also be why there were few reported predictions. Children’s predictions may be formulated internally and subsequently expressed as information-seeking (e.g. ‘Is it going to rain later?’) or action-taking (e.g. packing an umbrella). Thus, a shortcoming of our methodology is that it does not capture children’s potentially autonomous actions and thoughts when they are by themselves. It may be that our method particularly under-represents actions that older children, especially, might enact when a parent is not present.

### (c) Relations between our themes and adult functions of future thought

Our findings allow us to compare the possible functions of future-thinking in children with those proposed in adults and that we outlined in §1 [4,5,7,8]. We discuss each theme in the following sub-sections.

#### (i) Theme 1: expressing future desires and/or intentions

Theme 1 may mirror the adult function of intention formation and goal-setting [4,5,7]. It could be that children’s intentions are formed and expressed in reaction to their immediate environment. For example, one 4-year-old passed an arcade in the car and stated that ‘when he is a daddy’ he will live beside the arcade and go there every day with his kids. This intention may not have been expressed to eventually be implemented but, rather, because the child was unable to visit the arcade at that moment. Therefore, it may be that some children express future intentions as a way of communicating their existing wants that are unattainable in the present. Statements such as the previous one may serve as a form of present-oriented emotion regulation [8], whereby the child is reassuring himself that he will—eventually—be able to go to the arcade.

#### (ii) Theme 2: future-oriented information-seeking

Theme 2 may also relate to an emotion-regulation function [4,8]. Despite very few instances overall actually referring to children’s present or future emotions, a possible emotion regulation function is hinted at through the emotional valence of the events discussed by children. For example, enquiring about the occurrence of positive events (e.g. ‘When can we play Super Mario Brothers Wii again?’) may promote positive feelings in the present (e.g. reassurance [4]). In contrast, inquiring about potentially negative events (e.g. ‘Will there be skeletons at the museum?’) could be a way for children to prepare for adverse emotional reactions to future events [5].

Importantly, information-seeking is a potential function of future thought that we believe is more unique and prevalent in children, as compared with adults. This is because adults less often need to seek information from others to confirm what will be happening in their near, or distant, futures. However, children, who do not typically set or control their own schedules, need to seek information to be able to anticipate, predict and plan their future.

#### (iii) Theme 3: connecting present actions to future outcomes

Theme 3 may reflect the directive function of future-thinking that is ubiquitous in adults, whereby planning, problem-solving and regulating behaviour are enacted in service of our future goals [5,8]. Interestingly, even parents of 3-year-olds reported instances in this theme, which is at odds with the argument that such ‘planning’ emerges later in development [6,32].

#### (iv) Theme 4: predicting mental and physiological states

Theme 4 may reflect the adult functions of ‘self-continuity’ and ‘identity formation’ [8]. Through their predictions of what they will feel in the future, children may also be developing their sense of identity and self-continuity. It is unclear whether the predictions reported from our sample also influence children’s decision-making, as they do with adults [33,34]. Interestingly, this theme was minimally reflected—at least via parental report—in children’s natural environment despite previous findings showing improvements in this capacity between ages 3 and 5 (e.g. [26,27]). As we briefly discussed earlier, this may be due to predictions being generated internally and not expressed directly. Thus, it is possible that children do make predictions that inform decision-making, but these are not reflected in our data as predictions but instead as other themes such as taking action in the present. For example, a young child may have brought his toy on a long car ride because he had made a prediction that he would be bored.

### (d) Limitations and future directions

Any new methodological approach has its limitations, and ours is no exception. Most notably, because we have only gathered expressions of future thought observed by mothers, children’s underlying thought processes must be inferred by the researcher via the reports provided. As such, we cannot rule out the possibility that some instances, despite being convincing to the children’s mothers and the researchers, were not driven by any kind of mental projection into the future. Owing to this limitation, we can only speculate on the functional role that future-thinking plays in children’s daily lives. Furthermore, we refrained from developing sub-themes in our TA, owing to our small sample; however, future research should investigate additional dimensions of future thought in reported instances, such as temporal distance and episodicity. It is also important to recognize that our data come from a small sample (*N* = 12) and represent only a slice of time in children’s lives (i.e. one week). Therefore, our findings, particularly our quantitative analyses, should be interpreted with caution. Future naturalistic research with a larger sample and/or with a longitudinal design is necessary to continue to build on the data reported in this study.

Another important addition to our methodology would be to ‘train’ parents to identify the potential ‘triggers’ or cues of their children’s spontaneous future-thinking expressions; for example, noticing their child is using his Avengers water bottle when he asks to watch the Avengers movie when he is all grown up. Although we attempted to do so in the current study, parents in our sample did not appear to comprehend or recognize cues, even when they themselves stated one in the context description (e.g. a child asking to rent a specific movie from the library next time, while watching a movie that was rented from the library). It may be that the wording of our trigger question ‘Did you say or do anything …’ primed parents to examine their own behaviour, rather than the environment itself, as a trigger. Moreover, parents may have assumed that triggers needed to be very specific to the child’s statement (e.g. in the previous example, a cue related to the specific movie the child wanted to rent, rather than movies more broadly). Future iterations of this study should further train parents to identify environmental triggers/cues by giving them examples and encouraging them to do a ‘scan of their environment’ for cues relevant to what their child did and said.

Though our themes reflected three of the four proposed modes of future-thinking in adults [2], ‘simulation’ was notably absent. This may be because people’s (including children’s) simulations of the future are not often expressed, and hence parents would have had difficulty in identifying these. However, it is important to recognize that children’s simulations may have been inherent to all our themes [2]. Similar to our discussion on predictions, children may have simulated the future internally, which subsequently led to expressions of intentions and/or plans. It is also possible that information-seeking instances in theme 2 were expressed to lay the groundwork for simulations. For example, a child asking their parent about the upcoming day (e.g. ‘Will I go to daycare today?’) may lead to subsequent simulation of the day’s events.

Importantly, our methodology and findings can be built upon to create new experimental tasks. Through documenting children’s statements, we gain insight into how children express and conceptualize the future. For example, in our sample, some children denoted temporal distance between the present and a future event by counting ‘how many sleeps’. Some children also expressed the distant future as ‘when I’m a daddy/mommy’. This wording is different from that used in experimental tasks to prompt distant future-thinking; for example, Bélanger *et al.*'s future preference task [27] describes the distant future as ‘when you’re all grown up’. Incorporating the terminology used by children in their everyday lives may thus serve to increase their understanding of various experimental tasks. Our methodology also allows for a better understanding of the contexts, motivations and challenges that lead to children’s future-thinking expressions. This information can be used to inform new experimental tasks that provide an experience that is more reflective of children’s everyday lives. Finally, pairing our naturalistic approach with children’s performance on experimental tasks is also a fruitful future direction. For example, do those children whose parents report instances in theme 3 (‘connecting present actions to future outcomes’) also perform well on experimental tasks designed to assess directive future thought (e.g. spoon tasks)?

## 6. Conclusion

Naturalistic research is vital to understanding why children think about the future. Indeed, the developmental work in this area has leaned too heavily on experimental tasks that isolate future-thinking from the contexts in which they are typically enacted. In fact, if we reconsider Tulving’s spoon test [10], it is notable that it involved a young girl spontaneously engaging in a behaviour (i.e. retrieving a spoon) in her home to achieve a future goal. Although children in our study sometimes engaged in similar acts (e.g. bringing a toy on a long car ride), they also expressed intentions (which most often required parental support), sought information about the future and, sometimes, made predictions. Exploring future-thinking as it plays out in children’s daily lives will put researchers on both new theoretical and methodological paths that further elucidate the functional significance of mental time travel from its earliest emergence.

## Data Availability

All data and accompanying information have been uploaded into OSF [35].

## Footnotes

^1^
We acknowledge a lack of independence in our data because two of our parents are represented twice in the data for tracking two children each. We ran our quantitative analysis without two of these children (one sibling from each pair: a 3-year-old and a 6-year-old) and found that the loading across themes was similar.

^2^
We thank an anonymous reviewer for making the interesting point that, in ‘everyday life’, individual differences in future-thinking may be more pronounced than age-related ones.
